# Leveraging artificial intelligence to predict non-adherence to pediatric immunization schedules: a systematic review

**DOI:** 10.1007/s00431-026-07067-2

**Published:** 2026-05-15

**Authors:** Robin Rey, Arnaud G. L’Huillier, Laure F. Pittet

**Affiliations:** 1 Platform of Clinical Research in Pediatrics, Gynecology and Obstetrics, Department of Women, Children and Adolescent Health, Geneva University Hospitals, Geneva, Switzerland; 2 Pediatric Infectious Diseases Unit, Department of Woman, Children and Adolescent Health, Geneva University Hospitals, Geneva, Switzerland; 3 Unit of Immunology, Vaccinology, and Rheumatology, Division of General Pediatrics, Department of Women, Children and Adolescent Health, Geneva University Hospitals, Geneva, Switzerland

**Keywords:** Immunization, Pediatric, Artificial intelligence, Systematic review

## Abstract

Immunization is one of the most effective interventions to prevent infectious diseases. Identifying individuals at risk of non-adherence to immunization schedules could enable early interventions to improve coverage. With the increasing availability of large datasets with sociodemographic, medical, and geographic data, artificial intelligence (AI) offers new possibilities for identifying predictive patterns. We performed a systematic literature review of original studies that used AI to predict immunization non-adherence in children. Searches were conducted in PubMed, Embase, and Web of Science and no restrictions were applied regarding publication year, language, or study design. Study characteristics, dataset size, AI methods, performance metrics, and validation approaches were extracted. Risk of bias was assessed using PROBAST + AI. A total of 843 records were identified; after screening, 12 studies were included. Nine of 12 studies were conducted in low- and middle-income countries, used cross-sectional survey data, and analyzed vaccines from routine immunization schedules. Model performance was generally high; 9/12 studies reported an accuracy or an area under the receiver operating characteristic curve exceeding 80%, but no study conducted external validation. Predictors clustered into seven categories: maternal, paternal, family, child, geographic, wealth, and others. Among maternal and child-related factors, prenatal care and previous immunization history had a high predictive weight.

*Conclusion*: AI demonstrates a promising potential for identifying individuals at risk of vaccine non-adherence. However, the lack of external validation raises concerns about model overfitting and limits real-world applicability. Demographic health surveys were routinely repeated across countries and time periods and could therefore represent an ideal resource for external validation.

**Table:** 

**What is known:** • *Geographic, sociodemographic, and medical factors contribute to non-adherence to the immunization schedule. * • *Identifying these individuals can guide early interventions to improve immunization coverage.* **What is new:** • *Artificial intelligence can reveal unknown influential factors; prenatal care and delivery setting emerged as key factors in this systematic review.*

**Supplementary Information:**

The online version contains supplementary material available at https://doi.org/10.1007/s00431-026-07067-2.

## Introduction

Immunization is one of the most effective interventions to prevent infectious disease and one of the greatest successes in global healthcare history. According to the World Health Organization (WHO), more than 116 million infants are vaccinated annually, decreasing the mortality by 24% for infants under 5 years of age between 2010 and 2017 [1]. The Expanded Program on Immunization was launched by WHO 50 years ago, to improve availability of life-saving vaccines. It has successfully prevented 146 million deaths among children younger than 5 years [2].

Non-adherence to immunization schedule refers to all situations in which a child does not receive all the standard vaccines recommended for their age. It includes immunization dropout (children who receive the first dose(s) of a vaccine but fail to complete the required series) and “zero-dose” children (children who have not received any vaccine). Each year, nearly 20 million children fail to complete the full schedule of routine immunization, including 13 million “zero-dose” children [1]. These missed opportunities considerably increase the risk of outbreaks, and it is estimated that 700,000 children under 5 years of age die every year from vaccine-preventable disease [3].

Several factors such as geographic, sociodemographic, and medical factors contribute to non-adherence to the immunization schedule, and identifying individuals at risk is a key step to guide early interventions aiming at improving immunization coverage [4]. The dropout rate is higher in low- and lower-middle-income countries (12% and 5% respectively) for diphtheria-tetanus-pertussis (DTP) containing vaccines; these countries also account for 84% of the zero-dose children [4]. Sex of the child, poverty, maternal factors (such as age, marital status, and immunization history), delivery setting, prenatal and postnatal follow-up have been identified as predictive factors for non-adherence to the immunization schedule using traditional epidemiological approaches [5–7].

Artificial intelligence (AI) is a vast field with multiple methodologies and has revolutionized our capacity to extract actionable insights from complex datasets. It has therefore great potential for public health interventions for risk prediction, disease forecasting, and epidemic modeling [8–10]. With the increasing availability of large immunization datasets with sociodemographic, medical, and geographic data, AI offers novel possibilities for identifying predictive patterns of non-adherence.

The aim of this systematic review is (I) to evaluate the performance of AI-driven models in predicting non-adherence to immunization schedule in the pediatric population and (II) to summarize the predictors identified by AI models as contributing to non-adherence.

## Methodology

This study was conducted following the Preferred Reporting Items for Systematic Reviews and Meta-Analyses (PRISMA) guidelines (checklist is available in the Supplementary material 3).

### Research

Literature searches were done in PubMed, Embase, and Web of Science in July 2025, using the keywords “artificial intelligence”, “immunization”, “non-adherence”, and “factor” and their relevant synonyms combined with Boolean operators. Full search strategies are provided in Supplementary material 1. The reference lists of all included studies were also screened to identify additional eligible articles.

All original studies that used AI to predict immunization non-adherence in children were included, with no language restriction. Articles were excluded if the study did not include pediatric participants or in case of informal publications (commentary, letters, editorial, conference abstract). There was no restriction in the year of publication. One reviewer (R.R.) screened all articles first by titles and abstracts, and then in full texts. A second reviewer (L.P.) resolved uncertainties regarding articles selection at each stage of the process.

### Data

Data were extracted using a predefined form that captured the study population and data source, type of AI model, variables of the model, number of instances, performance, validation approach and interpretation of the predictive models. To streamline reporting, performance metrics were limited to the best-performing model for each study, which was generally the focus of subsequent interpretation. For the performance metrics, *accuracy* and *area under the curve* were extracted when available; otherwise, the *F1 score*, *sensitivity* and *specificity* were collected, along with their 95% interval confidence. The predictors frequently reported by studies were clustered into distinct categories.

### Risk of bias and synthesis methods

Risk of bias was assessed by one reviewer (R.R.) using the newly developed PROBAST + AI tool, designed specifically for evaluating AI-based prediction models [11]. Studies without evaluation of model performance were assessed only for the quality and applicability of the model-development part. Studies that included internal validation were also evaluated for risk of bias and applicability for validation. Findings for all the outcomes of interest are presented in descriptive tables; no meta-analysis was done because of the high heterogeneity across the included studies.

## Results

### Study selection

**Fig. 1:**
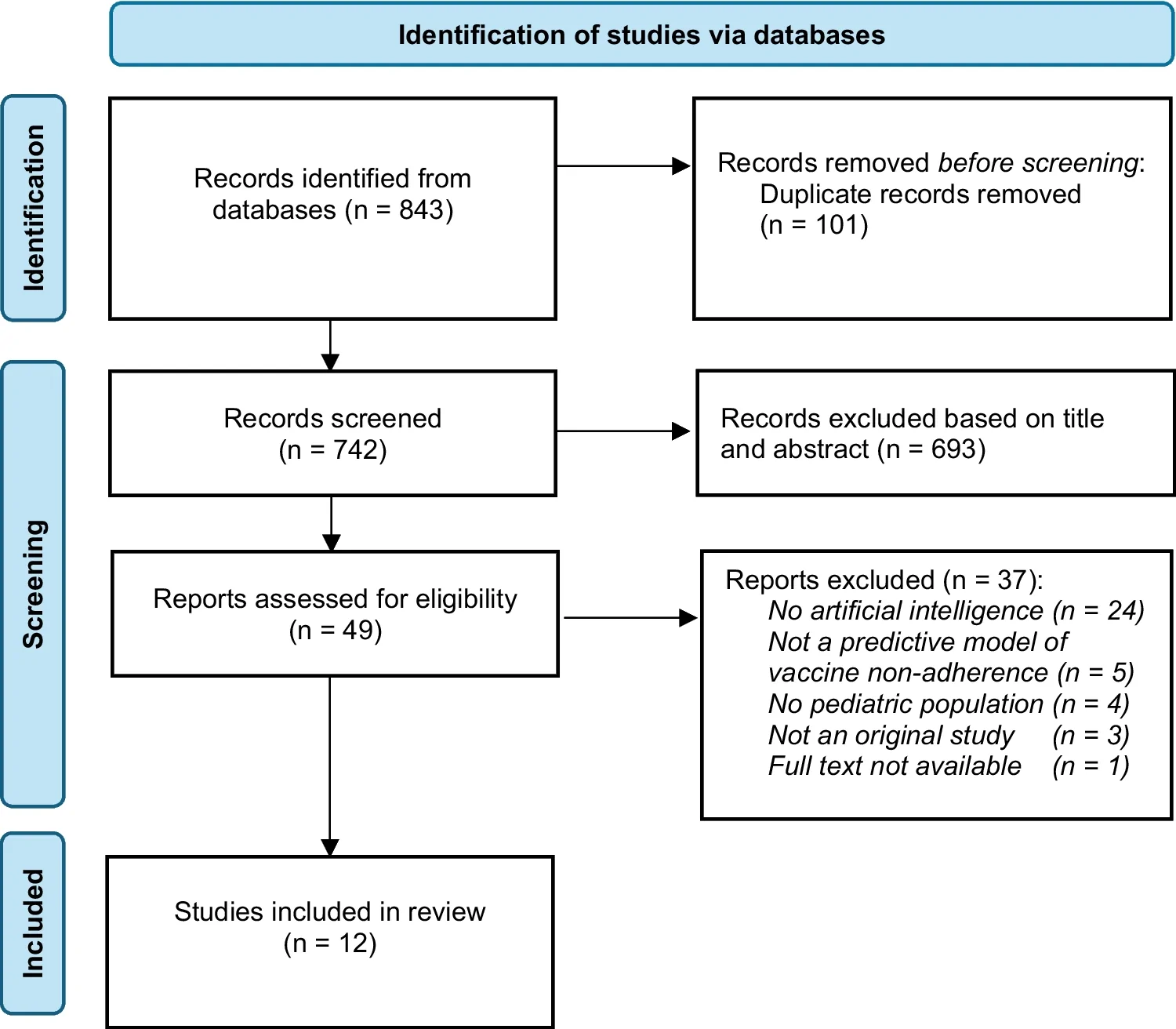
PRISMA flowchart of study records

A total of 843 publications were identified across the three databases. After removing 101 duplicates, 693 were excluded during title and abstract screening, and a further 37 after full-text review, resulting in 12 studies being included in the final selection (Fig. 1).

### Study characteristics

The characteristics of included studies and the performance of their algorithms are summarized in Table 1. All studies were published after 2018 and most (11/12) were conducted in low- and middle-income countries. Regarding the data sources, 9 of the 12 studies relied on cross-sectional Demographic and Health Survey (DHS) data [12–21], one used a registry [22], one used a retrospective cohort [23], and one used data from a randomized clinical trial [24]. Eleven studies focused on predicting non-adherence to routine immunization schedules, including Bacillus Calmette-Guérin (BCG), DTP, poliovirus, measles, and pneumococcal vaccines. One study focused on influenza vaccine, considered as an additional vaccine to routine immunization [23]. The AI approaches varied widely across studies, with no consistent methodological pattern. Model performance was generally high; 9/12 studies reported an accuracy or an area under the receiver operating characteristic curve (AUROC) exceeding 80% [13–19, 21, 23, 24]. Ten studies performed internal validation using data-splitting approaches, allocating 70 to 80% of observations for training and 30 to 20% for testing [13–19, 21–24]. Two studies reported no validation [12, 20]. No studies included external validation using data from a different setting or time period.

**Table 1:** This descriptive table resumes the characteristics of the included studies

N	Authors; year of publication	Data source; countries; number of instances or participants	Population; type of vaccines analyzed; predicted variable	Algorithm(s) used; validation of the model (% for validation/% for testing)	Performances of the best model (IC 95%)
**1**	**Abebe et al. (2020) (**12)	Cross-sectional (DHS; 2011) Ethiopia 8210 instances	Children (age not specified) Routine immunization ** Fully, partial, no vaccine	J48 DT, SMO, SVM, MLP, PART	J48 DT: ▪ Accuracy of 62% ▪ AUROC of 0.68
				No validation	
**2**	**Alemayehu et al. (2024) (**13)	Cross-sectional (DHS; 2016) Ethiopia 3893 instances	12- to 23-month old Measles Vaccine dropout	SVM, XGB, GBM, RF, DT, *k*-NN, NB, LR	XGB: ▪ Accuracy of 74% (95% CI; 72-75%) ▪ AUROC of 0.84
				Internal validation (70/30%)	
**3**	**Alemayehu et al. (2025) (**14)	Cross-sectional (DHS; year not specified) Ten East African countries 61,714 instances	Children (age not specified) Pentavalent III Vaccine dropout	Stacked ensemble machine learning model integrating 7 base learners: NB, GLM, DT, DL, RF, GBM, XGB	Stacked ensemble learner combining the weight of the 7 models with XGB: ▪ Accuracy of 94% ▪ AUC of 0.99
				Internal validation (70/30%)	
**4**	**Asnake et al. (2025) (**15)	Cross-sectional (DHS; 2022) Tanzania 2120 participants	12- to 23-month old Routine immunization** Zero-dose	LR, DT, RF, SVM, *k*-NN, XGB, NB	RF: ▪ Accuracy of 95% ▪ AUC of 0.99
				Internal validation (80/20%)	
**5**	**Biswas et al. (2023) (**16)	Repeated cross-sectional (DHS; 2006–2020) India, Mali and Nigeria 106,429 instances	12- to 23-month old DTP I Zero-dose	RC, DT, *k*-NN, MP	RC: ▪ F1 score of 0.84 ▪ Specificity of 0.54 ▪ Sensitivity of 0.77
				Internal validation (70/30%)	
**6**	**Chandir et al. (2018) (**22)	Registry (2012–2016) Pakistan 47,554 instances	Children (age not specified) Routine immunization* Incomplete	RF, RP, SVM, C-forest	RP: ▪ Accuracy of 79% ▪ AUC of 0.79 (95% CI; 78–80%)
				Internal validation (75/25%)	
**7**	**Nantongo et al. (2024) (**17)	Cross-sectional (DHS; 2016) Uganda 8929 instances for BCG 8482 for Pentavalent I 7976 for Pentavalent III 6793 for Measles	Children (age not specified) Routine immunization**** Incomplete	*k*-NN, DT, RF, SVM, NB, LR, XGB, AdaBoost, GB	RF (respectively for BCG, Pentavalent I, Pentavalent III, and Measles): ▪ Accuracy of 96%, 95%, 95%, and 87% ▪ AUROC of 0.88, 0.92, 0.98, and 0.88
				Internal validation (80/20%)	
**8**	** Qazi et al. (2020) ( **18) **and (2021) (**19)	Cross-sectional (DHS; 2017–2018) Pakistan 7153 instances	Children (age not specified) Routine immunization*** Fully vaccinated, partially high, partially medium, partially low, unvaccinated	DT, SVM, MLP, NB	MP: ▪ Accuracy of 98% ▪ AUC of 0.99
				Internal validation (70/30%)	
**9**	** Sameen et al. (2021) ( **24)	Community based cluster randomized clinical trial (Paigham-e-Sehat study; 2019) Pakistan 3113 instances	4 months old Routine immunization (details not available) Incomplete	LR, SVM, NB, DT, RF, ANN, XGB, *k*-NN, Kernel SVM	RF: ▪ Accuracy of 82% ▪ Sensitivity of 84% ▪ Specificity of 80%
				Internal validation (75/25%)	
**10**	**Santos et al. (2021) (**20)	Cross-sectional (DHS; 2010–2020) 92 low- and middle-income countries (Africa, Middle East, Europe, Asia, Pacific and Latin America) 210,509 participants	12- to 23-month-old Routine immunization** Zero-dose	CART	Not reported
				No validation	
**11**	**Shaham et al. (2020) (**23)	Retrospective cohort of electronic medical records (2011–2017) Israel 250,000 participants (1,553,907 instances)	Children and adults Influenza No seasonal immunization	LR, NB, XGB RF, light GBM RF, ANN	XGB: ▪ Accuracy of 90% ▪ AUROC of 0.88
				Internal validation (70/30%)	
**12**	**Tadese et al. (2024) (**21)	Cross-sectional (DHS; 2016–2021) Seven East African countries 27,806 participants	5 years old Routine immunization** Incomplete	LR, RF, *k*-NN, ANN, SVM, NB, XGB, DT	XGB: ▪ Accuracy of 79% ▪ AUROC of 0.86
				Internal validation (80/20%)	

*I* first dose, *II* second dose, *III* third dose, *ANN* artificial neural network, *CART* classification and regression tree, *DL* deep learning, *DHS* demographic and health survey, *DT* decision tree, *GB* gradient boosting, *GLM* generalized linear model, *k*-NN *k*-nearest neighbor, *LR* logistic regression, *MLP* multilayer perceptron, *NB* naïve Bayes, *PART* partial decision tree, *RC* ridge classification, *RF* random forest, *RP* recursive partitioning, *SMO* sequential minimal optimization, *SVM* support vector machine, *XGB* extreme gradient boosting

^*^BCG, pentavalent, measles

^**^BCG, polio, pentavalent or DTP, measles

^***^BCG, polio, pentavalent, measles, pneumococcal

^****^BCG, polio, pentavalent, measles, pneumococcal, rotavirus

**Fig. 2:**
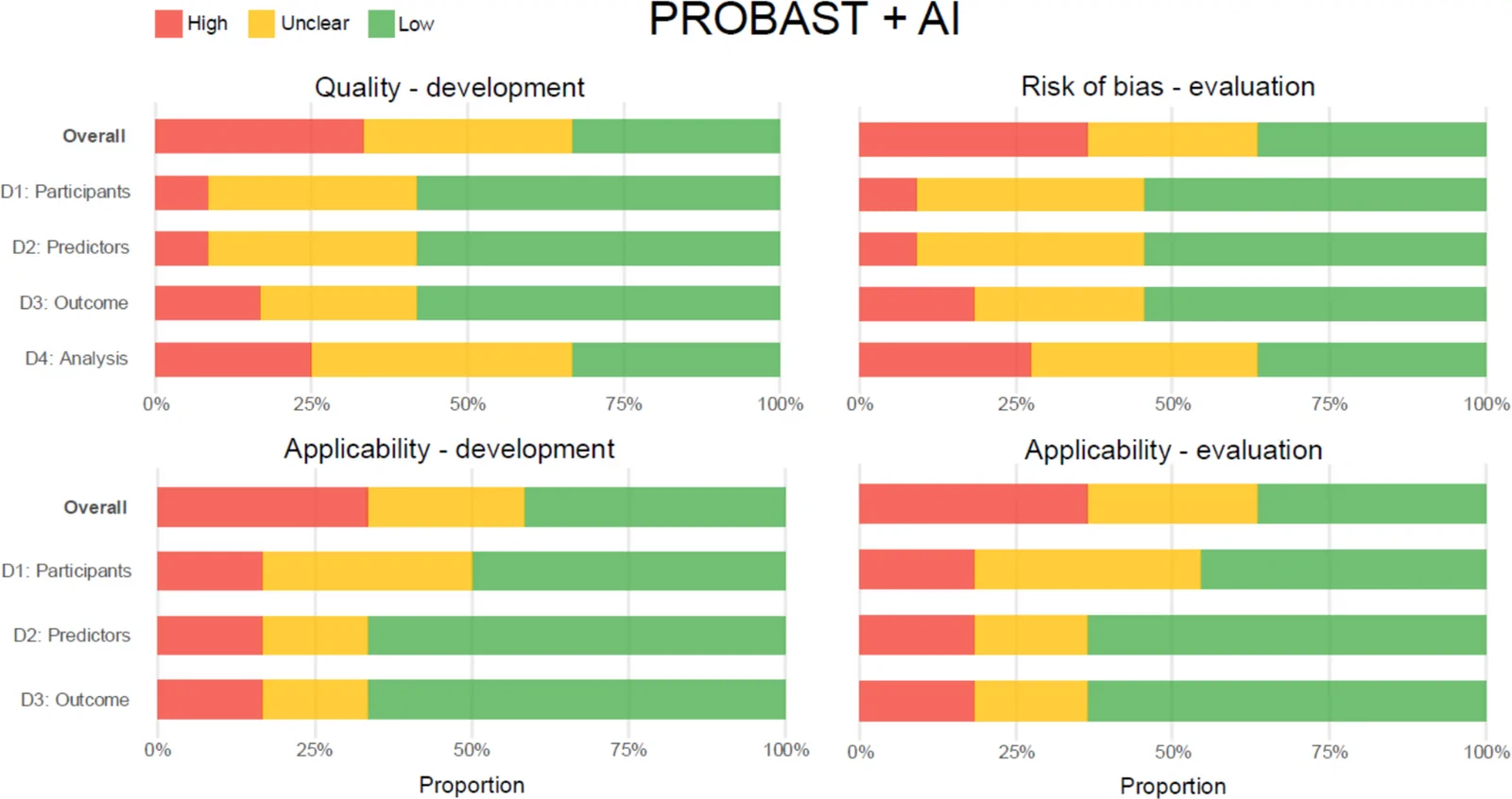
PROBAST + AI risk of bias assessment for included studies

The risk of bias assessed using the PROBAST + AI tool is shown in Fig. 2 and further detailed in Supplementary Material 2 (appendix). Regarding the development and validation of the models, data sources, data collection process and measurement were often insufficiently reported, particularly when derived from DHS data. The same limitation applied for handling missing data, censoring data and class imbalance (i.e., vaccinated/unvaccinated imbalance). Finally, immunization status was not always assessed in a similar way for all participants within a given model (e.g., based on immunization card or reported by parents). Conversely, model performance assessment along with predictor preprocessing and selection was generally well documented.

### Predictive factors and model interpretability

Table 2 summarizes the factors used to develop and validate each algorithm, along with the model-interpretability outputs, highlighting the strongest predictors of immunization non-adherence. The variables included in the models clustered into seven categories: maternal, paternal, family, child, geographic, wealth, and other factors (e.g., ethnicity, language, health insurance). Most studies combined variables from several of these categories to predict non-adherence. The most commonly used predictors were the household wealth index (11/12 studies), maternal age, maternal education, household size (each in 9/12), delivery setting, and region (each in 8/12). Many authors also reported model-interpretability results, indicating the relative contribution of each variable to the final prediction. Maternal factors generally carried greater predictive weight than paternal factors, and delivery setting was identified as a key predictor in half of the studies that included it (4/8). Few studies used prenatal follow-up (4/12) or previous immunization history (5/12) in their models; however, when included in the models, both these factors often had a high predictive weight (in 3/4 and 3/5 studies, respectively). Two commonly used factors never had any meaningful effect on the model performance: child sex (used in seven studies) and media exposure (used in five studies).

**Table 2:** Factors used to develop the algorithm of each study regrouped in seven categories.

	Abebe et al. (2020) [12]	Alemayehu et al. (2024) [13]	Alemayehu et al. (2025) [14]	Asnake et al. (2025) [15]	Biswas et al. (2023) [16]	Chandir et al. (2018) [22]	Nantongo et al. (2024) [17]	Qazi et al. (2020) [18] and (2021) [19]	Sameen et al. (2021) [24]	Santos et al. (2021) [20]	Shaham et al. (2020) [23]	Tadese et al. (2024) [21]
**Maternal**	**X**	**X**	**X**	**X**	**X**		**X**	**X**	**X**	**X**		**X**
Age	X	H	X	X			X	X	X	X		H
Education	X	H	X	H	X		X		X	X		X
Employment		H	X	H								
Prenatal follow up					H		X			H		H
**Paternal**	**X**	**X**	**X**	**X**			**X**		**X**			**X**
Age		H	X	X			X		X			
Education	X	H	X	X			X		H			X
**Family**	**X**	**X**	**X**	**X**	**X**		**X**	**X**		**X**		**X**
Marital status		X								X		X
Religion		H	X							X		
Household size	X	H	X	H	X		X	X		X		H
**Child**	**X**	**X**	**X**	**X**	**X**	**X**	**X**	**X**	**X**	**X**	**X**	**X**
Sex	X				X	X			X	X	X	X
Date of birth/age					X	X	X		X		H	
Delivery setting	X	X	H	X	H		X			H		H
Postnatal follow up		X	H	X								X
Previous vaccine						X	H	X	H		H	
**Geographic**	**X**	**X**	**X**	**X**	**X**	**X**	**X**	**X**	**X**			**X**
Region	X	H	X	X	X		H	X	X			
Rural/Urban	X	X	X		H	X		X				X
Healthcare accessibility	X	X	H	X								
**Wealth**	**X**	**X**	**X**	**X**	**X**		**X**	**X**	**X**	**X**	**X**	**X**
Wealth index	X	X	H	X	X		H	X	H	X	X	X
Media exposure	X	X	X							X		X
**Others** (e.g., ethnicity, language, health insurance)		**X**	**H**	**X**	**X**	**X**	**X**	**X**	**X**	**H**	**H**	**H**

The factors with an “H” have the highest impact on the prediction, as reported by each study

## Discussion

In this systematic review summarizing the performance of AI-based models to predict non-adherence to pediatric immunization schedule, accuracy and AUROC values superior to 80% were observed across studies, pointing to the potential of AI methodologies in this setting. A vast majority of the studies used information from low- and middle-income countries, which also have the lowest childhood immunization coverage [25, 26]. Studies used a mixed subset of predictors grouped in seven main categories, with maternal and pregnancy-related factors emerging as the most relevant.

The AI-based model helped to identify factors with the strongest impact on non-adherence and can guide future intervention aiming to improve immunization coverage [25, 27, 28]. For example, prenatal care and delivery settings emerged as key factors. This finding suggests that intervention targeting women delivering far from healthcare facilities, who may probably miss early counseling on immunization, could be particularly impactful. Several other maternal factors had a strong predictive value, advocating for women’s education. In contrast, some commonly presumed determinants, such as paternal age and education, or child’s sex and age, contributed less to the model performances, suggesting it may be less impactful to tailor interventions aimed at these factors. A review of more than 240 studies on inequalities in childhood vaccine coverage reported findings consistent with ours. Maternal education and economic status were the most frequently analyzed factors and 79% of the studies relied on DHS, Multiple Indicator Cluster Surveys (MICS), or other survey-based data sources [29].

A prior meta-analysis evaluating the coverage of routine childhood immunization and its variation across demographic and health characteristics in low- and middle-income countries using conventional statistical analysis, concluded that coverage varies with maternal education, birth order, and wealth status [25]. These three factors were not the most predominant in the present analysis, highlighting how AI-based methods may capture different heirarchies of predictors than those identified through traditional analyses. However, there is currently no evidence that AI identifies factors that would not have been detected using standard methods with an identical dataset.

Overall, the risk of bias and quality of the studies were consistent across domains, with the most robust studies being at low risk of bias throughout the assessment, and high-risk studies presenting concerns for the entire evaluation. A particularly relevant question of the PROBAST + AI evaluation tool is to assess how studies addressed class imbalance. As the outcome is non-adherence to immunization schedules, imbalances were observed across studies, since the proportion of children fully vaccinated was always significantly higher than that of those with incomplete immunization, dropouts, or zero-dose status. Because most children are vaccinated, prediction models may mainly learn to identify this majority group, which can reduce their ability to detect the smaller but clinically most relevant group of children who are not adherent to vaccination schedules. To address this issue, corrective strategies can be applied either at the data level, by reducing the number of fully vaccinated cases (undersampling) or by increasing the representation of non-adherent children using generated synthetic data. Methods can also be applied at the model level, by adjusting the decision threshold (by modifying the probability required to predict the non-vaccinated group) or by penalizing misclassification of non-adherent children more heavily [30–32].

The absence of external validation, using a dataset that differs in timing or location, is a major barrier to using these models for future predictions or customizing a regional approach for interventions. Without such validation, performances are likely overestimated and may decline substantially when models are applied in different settings, with varying data collection and population characteristics [10, 33]. As no studies validated or prospectively evaluated the performance of implemented AI systems, no conclusions can be drawn regarding their accuracy in predicting future immunization non-adherence. In addition, substantial heterogeneity was observed across studies in terms of vaccine types analyzed, algorithms used, predictors included, and definitions of non-adherence. These differences should be considered when interpreting the overall reported performance.

Many studies of this review used cross-sectional DHS data. As DHS surveys were routinely repeated across countries and time periods until the program was paused in 2025 [34], they represent an ideal resource for external validation of the AI-based model reported. As another potential source for external validation, the UNICEF MICS could be used as they now gather 260 datasets from 125 countries, including immunization-related data [35]. None of the studies included in our review used these datasets. Only one study used registry data as a source, despite the increasing implementation of electronic immunization registries (EIR) [36]. In the future of AI, we could imagine an integrated system into EIR to prospectively identify children at risk of future non-adherence, enabling targeted attention and counseling for these families.

Although exhaustive, the databases screened in this review are specific to medicine and not to computational sciences; other eligible articles may have been missed. Most of the included studies in this review were conducted in low- and middle-income countries. Other unexplored factors could be good predictors in other settings, such as vaccine hesitancy, profession of the parents, or exposure to vaccine-preventable infection, limiting the generalizability of these results.

## Conclusion

Our systematic review highlights the growing potential of AI to identify factors associated with non-adherence to the routine immunization schedule. Several studies offered meaningful interpretation, highlighting prenatal care and delivery setting as key elements associated with non-adherence that may help inform future interventions. However, the overall risk of bias was substantial, and the lack of external validation remains a critical barrier to the reliable use of these models in clinical or public health practice.

## Supplementary Information

Below is the link to the electronic supplementary material.

ESM1 (DOCX 45.3 KB)

## Data Availability

No datasets were generated or analysed during the current study.

## References

[1] (2024) Immunization agenda 2030: a global strategy to leave no one behind. Vaccine 42:S5–14. 10.1016/j.vaccine.2022.11.042 39004466

[2] Shattock AJ, Johnson HC, Sim SY, Carter A, Lambach P, Hutubessy RCW et al (2024) Contribution of vaccination to improved survival and health: modelling 50 years of the Expanded Programme on Immunization. Lancet 403:2307–16. 10.1016/S0140-6736(24)00850-X PMC11140691 38705159

[3] Frenkel LD (2021) The global burden of vaccine-preventable infectious diseases in children less than 5 years of age: implications for COVID-19 vaccination. How can we do better? Allergy Asthma Proc 42:378–385. 10.2500/aap.2021.42.210065 PMC8677503 34474707

[4] Kaur G (2023) Routine vaccination coverage — worldwide, 2022. MMWR Morb Mortal Wkly Rep. 10.15585/mmwr.mm7243a1 PMC10602616 37883326

[5] Biks GA, Shiferie F, Tsegaye DA, Asefa W, Alemayehu L, Wondie T et al (2024) High prevalence of zero-dose children in underserved and special setting populations in Ethiopia using a generalize estimating equation and concentration index analysis. BMC Public Health 24:592. 10.1186/s12889-024-18077-w PMC10893596 38395877

[6] Kassahun MB, Biks GA, Teferra AS (2015) Level of immunization coverage and associated factors among children aged 12–23 months in Lay Armachiho District, North Gondar Zone, Northwest Ethiopia: a community based cross sectional study. BMC Res Notes 8:239. 10.1186/s13104-015-1192-y PMC4467673 26071403

[7] Ali Y, Mekonnen FA, Molla Lakew A, Wolde HF (2020) Poor maternal health service utilization associated with incomplete vaccination among children aged 12-23 months in Ethiopia. Hum Vaccin Immunother 16:1202–7. 10.1080/21645515.2019.1670124 PMC7227678 31545118

[8] Panteli D, Adib K, Buttigieg S, Goiana-da-Silva F, Ladewig K, Azzopardi-Muscat N et al (2025) Artificial intelligence in public health: promises, challenges, and an agenda for policy makers and public health institutions. Lancet Public Health Elsevier 10:e428–e432. 10.1016/S2468-2667(25)00036-2 PMC12040707 40031938

[9] Olawade DB, Wada OJ, David-Olawade AC, Kunonga E, Abaire O, Ling J (2023) Using artificial intelligence to improve public health: a narrative review. Front Public Health 11. 10.3389/fpubh.2023.1196397 PMC10637620 37954052

[10] Hunter DJ, Holmes C (2023) Where medical statistics meets artificial intelligence. N Engl J Med 389:1211–1219. 10.1056/NEJMra2212850 37754286

[11] Moons KGM, Damen JAA, Kaul T, Hooft L, Navarro CA, Dhiman P et al (2025) PROBAST+AI: an updated quality, risk of bias, and applicability assessment tool for prediction models using regression or artificial intelligence methods. BMJ. 10.1136/bmj-2024-082505 PMC11931409 40127903

[12] Abebe H, Meshesha M (2020) Describing infant immunization status in Ethiopian. IOSR J Nurs Health Sci 9:01–06. 10.9790/1959-0901090106

[13] Alemayehu MA (2024) Machine learning algorithms for prediction of measles one vaccination dropout among 12–23 months children in Ethiopia. BMJ Open 14:e089764. 10.1136/bmjopen-2024-089764 PMC11575239 39542486

[14] Alemayehu MA, Kebede SD, Walle AD, Mamo DN, Enyew EB, Adem JB (2025) A stacked ensemble machine learning model for the prediction of pentavalent 3 vaccination dropout in East Africa. Front Big Data. 10.3389/fdata.2025.1522578 PMC12009798 40260052

[15] Asnake AA, Seifu BL, Gebrehana AK (2025) Prediction of zero-dose children using supervised machine learning algorithm in Tanzania: evidence from the recent 2022 Tanzania demographic and health survey. BMJ Open 15:e097395. 10.1136/bmjopen-2024-097395 PMC11931888 40122556

[16] Biswas A, Tucker J, Bauhoff S (2023) Performance of predictive algorithms in estimating the risk of being a zero-dose child in India, Mali and Nigeria. BMJ Glob Health 8:e012836. 10.1136/bmjgh-2023-012836 PMC10583101 37821114

[17] Nantongo BA, Nabukenya J, Nabende P, Kamulegeya J (2024) A retrospective cohort study on predicting infants at a risk of defaulting routine immunization in Uganda using machine learning models. JAMIA Open 7:ooae132. 10.1093/jamiaopen/ooae132 PMC11645499 39677537

[18] Qazi S, Usman M, Mahmood A, Abbasi A, Attique M, Nam Y (2020) Smart healthcare using data-driven prediction of immunization defaulters in expanded program on immunization (EPI). Comput Mater Contin 66:589–602. 10.32604/cmc.2020.012507

[19] Qazi S, Usman M, Mahmood A (2021) A data-driven framework for introducing predictive analytics into expanded program on immunization in Pakistan. Wien Klin Wochenschr 133:695–702. 10.1007/s00508-020-01737-3 32945946

[20] Santos TM, Cata-Preta BO, Victora CG, Barros AJD (2021) Finding children with high risk of non-vaccination in 92 low- and middle-income countries: a decision tree approach. Vaccines 9:646. 10.3390/vaccines9060646 PMC8231774 34199179

[21] Tadese ZB, Nigatu AM, Yehuala TZ, Sebastian Y (2024) Prediction of incomplete immunization among under-five children in East Africa from recent demographic and health surveys: a machine learning approach. Sci Rep 14:11529. 10.1038/s41598-024-62641-8 PMC11109113 38773175

[22] Chandir S, Siddiqi DA, Hussain OA, Niazi T, Shah MT, Dharma VK et al (2018) Using predictive analytics to identify children at high risk of defaulting from a routine immunization program: feasibility study. JMIR Public Health Surveill 4:e63. 10.2196/publichealth.9681 PMC6231754 30181112

[23] Shaham A, Chodick G, Shalev V, Yamin D (2020) Personal and social patterns predict influenza vaccination decision. BMC Public Health 20:222. 10.1186/s12889-020-8327-3 PMC7017468 32050948

[24] Sameen F, Kazi A, Kazmi M, Abbasi M, Qazi S, Stergioulas L (2021) Improving routine immunization coverage through optimally designed predictive models. Comput Mater Contin 70:375–395. 10.32604/cmc.2022.019167

[25] Tamir TT, Tekeba B, Techane MA, Alemu TG, Wubneh CA, Wassie YA (2025) Coverage of complete basic childhood vaccination and its variation by basic characteristics among children aged 12-23 months in 41 low- and middle-income countries: a meta-analysis of demographic and health survey reports between 2015 and 2025. Vaccine 54:127019. 10.1016/j.vaccine.2025.127019 40081230

[26] Lai X, Zhang H, Pouwels KB, Patenaude B, Jit M, Fang H (2023) Estimating global and regional between-country inequality in routine childhood vaccine coverage in 195 countries and territories from 2019 to 2021: a longitudinal study. eClinicalMedicine 60:102042. 10.1016/j.eclinm.2023.102042 PMC10249397 37304497

[27] Oyo-Ita A, Oduwole O, Arikpo D, Effa EE, Esu EB, Balakrishna Y et al (2023) Interventions for improving coverage of childhood immunisation in low‐ and middle‐income countries - Oyo-Ita, A - | Cochrane Library. [cited 2025 Nov 13]; https://www.cochranelibrary.com/cdsr/doi/10.1002/14651858.CD008145.pub4/full. Accessed 13 Nov 2025 PMC10698843 38054505

[28] Siddiqui FA, Padhani ZA, Salam RA, Aliani R, Lassi ZS, Das JK et al (2022) Interventions to improve immunization coverage among children and adolescents: a meta-analysis. Pediatrics 149:e2021053852D. 10.1542/peds.2021-053852D 35503337

[29] Lyons C, Nambiar D, Johns NE, Allorant A, Bergen N, Hosseinpoor AR (2024) Inequality in childhood immunization coverage: a scoping review of data sources, analyses, and reporting methods. Vaccines 12:850. 10.3390/vaccines12080850 PMC11360733 39203976

[30] Buda M, Maki A, Mazurowski MA (2018) A systematic study of the class imbalance problem in convolutional neural networks. Neural Netw 106:249–259. 10.1016/j.neunet.2018.07.011 30092410

[31] Li D-C, Hu SC, Lin L-S, Yeh C-W (2017) Detecting representative data and generating synthetic samples to improve learning accuracy with imbalanced data sets. PLoS One 12:e0181853. 10.1371/journal.pone.0181853 PMC5542532 28771522

[32] Esposito C, Landrum GA, Schneider N, Stiefl N, Riniker S (2021) GHOST: adjusting the decision threshold to handle imbalanced data in machine learning. J Chem Inf Model 61:2623–2640. 10.1021/acs.jcim.1c00160 34100609

[33] Kelly CJ, Karthikesalingam A, Suleyman M, Corrado G, King D (2019) Key challenges for delivering clinical impact with artificial intelligence. BMC Med 17:195. 10.1186/s12916-019-1426-2 PMC6821018 31665002

[34] Nott J, Kaunda-Khangamwa B, Mathanga DP, Molenaar J, Taithe B, Phiri C et al (2026) Beyond the demographic and health survey: on the past and future of population health surveillance. BMJ Glob Health. 10.1136/bmjgh-2025-022023 PMC12918669 41708140

[35] Surveys [Internet]. [cited 2026 Apr 27]. https://mics.unicef.org/surveys. Accessed 27 Apr 2026

[36] Vigezzi GP, Maggioni E, Clavario L, Clerico Mosina L, Raso E, Marjin C et al (2025) Immunization information systems’ implementation and characteristics across the world: a systematic review of the literature. Expert Rev Vaccines 24:668–702. 10.1080/14760584.2025.2510338 40413630

