# Supplementary material for: Leveraging artificial intelligence to predict non-adherence to pediatric immunization schedules: a systematic review
Source: Eur J Pediatr. 2026 May 15;185(6):396. doi: 10.1007/s00431-026-07067-2 (PMC13652894; doi:10.1007/s00431-026-07067-2)
Supplement: Supplementary file 1 [file 431_2026_7067_MOESM1_ESM.docx]

**Appendix 1: Research strategy**

PubMed: 242 articles

("artificial intelligence"[MeSH Terms] OR "machine learning"[MeSH Terms] OR "deep learning"[MeSH Terms] OR "neural networks, computer"[MeSH Terms] OR "natural language processing"[MeSH Terms] OR "data mining"[MeSH Terms] OR "decision support techniques"[MeSH Terms] OR "Algorithms"[MeSH] OR "artificial intelligence" OR "machine learning" OR "deep learning" OR "neural network" OR "natural language processing" OR "data mining" OR "predictive model" OR "prediction model" OR algorithm* OR AI OR "computer-assisted" OR "computerised model" OR "computational model" OR "supervised learning" OR "unsupervised learning" OR "ensemble learning" OR "random forest" OR "support vector machine" OR "SVM" OR "decision tree" OR "naive bayes" OR "k-nearest neighbor")

AND ("immunization programs"[MeSH Terms] OR "immunization"[MeSH Terms] OR "vaccination"[MeSH Terms] OR "mass vaccination"[MeSH Terms] OR "Vaccination coverage"[MeSH] OR "Vaccination refusal"[MeSH] OR immunizat* OR immunisat* OR vaccin*)

AND (default* OR dropout* OR "missed vaccination" OR "missed immunization" OR "missed doses" OR "non-adherence" OR "non compliance" OR "loss to follow-up" OR "lost to follow-up" OR "incomplete vaccination" OR "partial vaccination" OR "under-vaccinated" OR "underimmunized" OR "under immunised" OR "zero-dose" OR "vaccination delay" OR "immunization delay" OR "vaccine refusal" OR "immunization refusal" OR "vaccination gap" OR "immunization gap" OR "coverage gap" OR "under-vaccinated" OR "under-immunized" OR "incompletely vaccinated" OR "incompletely immunized" OR "Medication Adherence"[MeSH] OR "Patient Compliance"[MeSH])

AND (factor* OR predict* OR determinant* OR variable* OR risk* OR correlat* OR characteristic* OR association* OR indicator*)

Embase: 509 articles

('artificial intelligence'/exp OR 'machine learning'/exp OR 'deep learning'/exp OR 'artificial neural network'/exp OR 'natural language processing'/exp OR 'data mining'/exp OR 'decision support system'/exp OR 'algorithm'/exp OR 'artificial intelligence' OR 'machine learning' OR 'deep learning' OR 'neural network' OR 'natural language processing' OR 'data mining' OR 'predictive model' OR 'prediction model' OR 'algorithm*' OR 'ai' OR 'computer-assisted' OR 'computerised model' OR 'computational model' OR 'supervised learning' OR 'unsupervised learning' OR 'ensemble learning' OR 'random forest' OR 'support vector machine' OR 'svm' OR 'decision tree' OR 'naive bayes' OR 'k-nearest neighbor')

AND ('preventive health service'/exp OR 'immunization'/exp OR 'vaccination'/exp OR 'mass immunization'/exp OR 'vaccination coverage'/exp OR 'vaccination refusal'/exp OR 'immunizat*' OR 'immunisat*' OR 'vaccin*')

AND ('default*' OR 'dropout*' OR 'missed vaccination' OR 'missed immunization' OR 'missed doses' OR 'non-adherence' OR 'non compliance' OR 'loss to follow-up' OR 'lost to follow-up' OR 'incomplete vaccination' OR 'partial vaccination' OR 'underimmunized' OR 'under immunised' OR 'zero-dose' OR 'vaccination delay' OR 'immunization delay' OR 'vaccine refusal' OR 'immunization refusal' OR 'vaccination gap' OR 'immunization gap' OR 'coverage gap' OR 'under-vaccinated' OR 'under-immunized' OR 'incompletely vaccinated' OR 'incompletely immunized' OR 'medication compliance'/exp OR 'patient compliance'/exp)

AND ('factor*' OR 'predict*' OR 'determinant*' OR 'variable*' OR 'risk*' OR 'correlat*' OR 'characteristic*' OR 'association*' OR 'indicator*')

Web of Science: 92 articles

ALL=("artificial intelligence" OR "machine learning" OR "deep learning" OR "neural network" OR "natural language processing" OR "data mining" OR "decision support techniques" OR "algorithm" OR "predictive model" OR "prediction model" OR algorithm* OR AI OR "computer-assisted" OR "computerised model" OR "computational model" OR "supervised learning" OR "unsupervised learning" OR "ensemble learning" OR "random forest" OR "support vector machine" OR SVM OR "decision tree" OR "naive bayes" OR "k-nearest neighbor")

AND ALL=("immunization program" OR "immunization" OR "vaccination" OR "mass vaccination" OR "vaccination coverage" OR "vaccination refusal" OR immunizat* OR immunisat* OR vaccin*)

AND ALL=(default* OR dropout* OR "missed vaccination" OR "missed immunization" OR "missed doses" OR "non-adherence" OR "non compliance" OR "loss to follow-up" OR "lost to follow-up" OR "incomplete vaccination" OR "partial vaccination" OR "under-vaccinated" OR "underimmunized" OR "under immunised" OR "zero-dose" OR "vaccination delay" OR "immunization delay" OR "vaccine refusal" OR "immunization refusal" OR "vaccination gap" OR "immunization gap" OR "coverage gap" OR "under-vaccinated" OR "under-immunized" OR "incompletely vaccinated" OR "incompletely immunized" OR "Medication Adherence" OR "Patient Compliance")

AND ALL=(factor* OR predict* OR determinant* OR variable* OR risk* OR correlat* OR characteristic* OR association* OR indicator*)

**Appendix 2: PROBAST + AI**

|  |  | Quality - Development | | | | | Applicability - Development | | | | Risk of bias - Evaluation | | | | | Applicability - Evaluation | | | |
| --- | --- | --- | --- | --- | --- | --- | --- | --- | --- | --- | --- | --- | --- | --- | --- | --- | --- | --- | --- |
|  |  | **Overall** | Participants | Predictors | Outcome | Analysis | **Overall** | Participants | Predictors | Outcome | **Overall** | Participants | Predictors | Outcome | Analysis | **Overall** | Participants | Predictors | Outcome |
| Abebe et al., 2020 (12) | | **H** | U | L | L | H | **U** | U | L | L | **H** | U | L | L | H | **U** | U | L | L |
| Alemayehu et al., 2024 (13) | | **L** | L | L | L | L | **L** | L | L | L | **L** | L | L | L | L | **L** | L | L | L |
| Alemayehu et al., 2025 (14) | | **L** | L | L | L | L | **L** | L | L | L | **L** | L | L | L | L | **L** | L | L | L |
| Asnake et al., 2025 (15) | | **L** | L | L | L | L | **L** | L | L | L | **L** | L | L | L | L | **L** | L | L | L |
| Biswas et al., 2023 (16) | | **U** | U | U | U | U | **H** | U | U | H | **U** | U | U | U | U | **H** | U | U | H |
| Chandir et al., 2018 (22) | | **H** | H | H | U | H | **H** | H | H | U | **H** | H | H | U | H | **H** | H | H | U |
| Nantongo et al., 2024 (17) | | **H** | L | U | H | U | **U** | L | U | U | **H** | L | U | H | U | **U** | L | U | U |
| Qazi et al. 2020 (18) and 2021 (19) | | **H** | U | U | H | H | **H** | U | L | H | **H** | U | U | H | H | **H** | U | L | H |
| Sameen et al. 2022 (24) | | **U** | U | L | L | U | **U** | U | L | L | **U** | U | L | L | U | **U** | U | L | L |
| Santos et al., 2021 (20) | | **U** | L | L | L | U | **L** | L | L | L | **NA** | NA | NA | NA | NA | **NA** | NA | NA | NA |
| Shaham et al., 2020 (23) | | **U** | L | U | U | U | **H** | H | H | L | **U** | L | U | U | U | **H** | H | H | L |
| Tadese et al., 2024 (21) | | **L** | L | L | L | L | **L** | L | L | L | **L** | L | L | L | L | **L** | L | L | L |

Table 3: This table shows the details for each study of the risk of bias assessment with the PROBAST+AI tool. H: High; L: Low; U: Unclear; NA: Not applicable.

**Appendix 3 : PRISMA checklist**

| **Section and Topic** | **Item #** | **Checklist item** | **Location where item is reported** |
| --- | --- | --- | --- |
| **TITLE** | | |  |
| Title | 1 | Identify the report as a systematic review. | p.1 |
| **ABSTRACT** | | |  |
| Abstract | 2 | See the PRISMA 2020 for Abstracts checklist. | p.1 |
| **INTRODUCTION** | | |  |
| Rationale | 3 | Describe the rationale for the review in the context of existing knowledge. | p.3 |
| Objectives | 4 | Provide an explicit statement of the objective(s) or question(s) the review addresses. | p.3 |
| **METHODS** | | |  |
| Eligibility criteria | 5 | Specify the inclusion and exclusion criteria for the review and how studies were grouped for the syntheses. | p.4 |
| Information sources | 6 | Specify all databases, registers, websites, organisations, reference lists and other sources searched or consulted to identify studies. Specify the date when each source was last searched or consulted. | p.3 |
| Search strategy | 7 | Present the full search strategies for all databases, registers and websites, including any filters and limits used. | Appendix 1 |
| Selection process | 8 | Specify the methods used to decide whether a study met the inclusion criteria of the review, including how many reviewers screened each record and each report retrieved, whether they worked independently, and if applicable, details of automation tools used in the process. | p.4 |
| Data collection process | 9 | Specify the methods used to collect data from reports, including how many reviewers collected data from each report, whether they worked independently, any processes for obtaining or confirming data from study investigators, and if applicable, details of automation tools used in the process. | p.4 |
| Data items | 10a | List and define all outcomes for which data were sought. Specify whether all results that were compatible with each outcome domain in each study were sought (e.g. for all measures, time points, analyses), and if not, the methods used to decide which results to collect. | p.4 |
|  | 10b | List and define all other variables for which data were sought (e.g. participant and intervention characteristics, funding sources). Describe any assumptions made about any missing or unclear information. | p.4 |
| Study risk of bias assessment | 11 | Specify the methods used to assess risk of bias in the included studies, including details of the tool(s) used, how many reviewers assessed each study and whether they worked independently, and if applicable, details of automation tools used in the process. | p.4 |
| Effect measures | 12 | Specify for each outcome the effect measure(s) (e.g. risk ratio, mean difference) used in the synthesis or presentation of results. | p.4 |
| Synthesis methods | 13a | Describe the processes used to decide which studies were eligible for each synthesis (e.g. tabulating the study intervention characteristics and comparing against the planned groups for each synthesis (item #5)). | p.4 |
|  | 13b | Describe any methods required to prepare the data for presentation or synthesis, such as handling of missing summary statistics, or data conversions. | NA |
|  | 13c | Describe any methods used to tabulate or visually display results of individual studies and syntheses. | p.4 |
|  | 13d | Describe any methods used to synthesize results and provide a rationale for the choice(s). If meta-analysis was performed, describe the model(s), method(s) to identify the presence and extent of statistical heterogeneity, and software package(s) used. | p.4 |
|  | 13e | Describe any methods used to explore possible causes of heterogeneity among study results (e.g. subgroup analysis, meta-regression). | NA |
|  | 13f | Describe any sensitivity analyses conducted to assess robustness of the synthesized results. | NA |
| Reporting bias assessment | 14 | Describe any methods used to assess risk of bias due to missing results in a synthesis (arising from reporting biases). | NA |
| Certainty assessment | 15 | Describe any methods used to assess certainty (or confidence) in the body of evidence for an outcome. | NA |
| **RESULTS** | | |  |
| Study selection | 16a | Describe the results of the search and selection process, from the number of records identified in the search to the number of studies included in the review, ideally using a flow diagram. | p.5 |
|  | 16b | Cite studies that might appear to meet the inclusion criteria, but which were excluded, and explain why they were excluded. | p.5 |
| Study characteristics | 17 | Cite each included study and present its characteristics. | p.5-6-7 |
| Risk of bias in studies | 18 | Present assessments of risk of bias for each included study. | p.7 – Appendix 2 |
| Results of individual studies | 19 | For all outcomes, present, for each study: (a) summary statistics for each group (where appropriate) and (b) an effect estimate and its precision (e.g. confidence/credible interval), ideally using structured tables or plots. | p.6-7 |
| Results of syntheses | 20a | For each synthesis, briefly summarise the characteristics and risk of bias among contributing studies. | p.7 |
|  | 20b | Present results of all statistical syntheses conducted. If meta-analysis was done, present for each the summary estimate and its precision (e.g. confidence/credible interval) and measures of statistical heterogeneity. If comparing groups, describe the direction of the effect. | NA |
|  | 20c | Present results of all investigations of possible causes of heterogeneity among study results. | NA |
|  | 20d | Present results of all sensitivity analyses conducted to assess the robustness of the synthesized results. | NA |
| Reporting biases | 21 | Present assessments of risk of bias due to missing results (arising from reporting biases) for each synthesis assessed. | NA |
| Certainty of evidence | 22 | Present assessments of certainty (or confidence) in the body of evidence for each outcome assessed. | NA |
| **DISCUSSION** | | |  |
| Discussion | 23a | Provide a general interpretation of the results in the context of other evidence. | p.9 |
|  | 23b | Discuss any limitations of the evidence included in the review. | p.9-10 |
|  | 23c | Discuss any limitations of the review processes used. | p.9-10 |
|  | 23d | Discuss implications of the results for practice, policy, and future research. | p.9-10 |
| **OTHER INFORMATION** | | |  |
| Registration and protocol | 24a | Provide registration information for the review, including register name and registration number, or state that the review was not registered. | NA |
|  | 24b | Indicate where the review protocol can be accessed, or state that a protocol was not prepared. | NA |
|  | 24c | Describe and explain any amendments to information provided at registration or in the protocol. | NA |
| Support | 25 | Describe sources of financial or non-financial support for the review, and the role of the funders or sponsors in the review. | NA |
| Competing interests | 26 | Declare any competing interests of review authors. | NA |
| Availability of data, code and other materials | 27 | Report which of the following are publicly available and where they can be found: template data collection forms; data extracted from included studies; data used for all analyses; analytic code; any other materials used in the review. | Appendix |
